# Spatial Distribution of Leprosy in the Amazon Region of Brazil 

**DOI:** 10.3201/eid1504.081378

**Published:** 2009-04

**Authors:** Maria L.F. Penna, Maria L. Wand-del-Rey de Oliveira, Gerson Penna

**Affiliations:** Federal Ministry of Health, Rio de Janeiro, Brazil (M.L.F. Penna); Federal University of Rio de Janeiro Medical School, Rio de Janeiro (M.L.W.d.R.de Oliveira); University of Brasília, Brasília, Brazil (G. Penna)

**Keywords:** Leprosy, Amazon, Brazil, cluster analysis, detection rate, disease control, epidemiology, dispatch

## Abstract

To detect areas with increased case-detection rates, we used spatial scan statistics to identify 5 of 10 clusters of leprosy in the Amazon region of Brazil. Despite increasing economic development, population growth, and road infrastructure, leprosy is endemic to this region, which is a source of case exportation to other parts of Brazil.

Leprosy is a public health problem in Brazil. Despite economic development, expansion of public healthcare, and efforts of the leprosy control program in the past 30 years, this disease has not been eliminated, and new cases are still being detected.

Leprosy has been a notifiable disease in Brazil since 1980. In 1999, a leprosy surveillance system was adopted throughout the country. Each reported case is recorded by the municipal health authority into its database. This information is then reported to the Ministry of Heath. The leprosy control program in Brazil distributes free dapsone, rifampin, and clofazimine as part of the World Health Organization multidrug regimen for treatment of leprosy. The need for treatment is determined on the basis of reported data. Brazil has 5,560 municipalities, 26 states, and 1 Federal District, and an area of 8,514,205 km^2^. Knowledge of the spatial distribution of leprosy will increase the efficiency of the leprosy control program in this country.

Leprosy has been highly endemic to the Amazon region of Brazil for >100 years. In the 19th century, leprosy incidence was high among Native Americans in the state of Pará (*1*). In 1913, Oswaldo Cruz, then head of the Brazilian Public Health Division, recognized the high frequency of leprosy in the Amazon River Basin (*2*). In 1975, Agrícola (*3*) reported that state of Acre, in the western Amazon region of Brazil, had the highest seroprevalence rate for leprosy. Conversely, states in northeastern Brazil, which have a semiarid climate, had the lowest seroprevalence rates. In 2007, the state of Mato Grosso in the southern Amazon region of Brazil, reported the highest case-detection rate (100.27/100,000 inhabitants), and the state of Rio Grande do Sul, the southernmost state, reported the lowest case-detection rate (1.74/100,000 inhabitants) (*4*). These findings suggest that the spatial distribution of leprosy has changed in the past 30 years.

## The Study

To detect areas with an increased case-detection rate for leprosy, we used spatial scan statistics (*5*). This method scans an area for leprosy clusters without a priori knowledge of their location or size. A circular window moves though a map with its center at the coordinates of municipal councils. At each position, the radius of the circular window varies from 0 km to 500 km, and each window includes different groups of neighboring municipalities. A Poisson model defines the presence of spatial clusters. Under the null hypothesis, the expected number of cases in each area is proportional to the person-years in that area. All possible clusters are tested for statistical significance by a log likelihood ratio test, which accounts for multiple testing (*6*). The log likelihood ratio defines cluster order.

We used Satscan software (*7*) to obtain statistical estimates on the basis of the number of new cases diagnosed during 2005–2007 by municipality of residence (obtained from the national database available on July 5, 2008) and population estimates by municipality for the same period (obtained from the Brazilian Institute of Geography and Statistics). The 10 most probable nonoverlapping clusters of leprosy (Table 1) were located between latitudes 21°S and 4°N. These clusters comprised 1,173 municipalities with 65,357 cases diagnosed during 2005–2007, 53.5% of all cases in Brazil, and 17% of the person-years in this period (33,080,363 inhabitants in 2007). These clusters covered a wide but sparsely populated area (Figure). The leprosy case-detection rate for the clusters was 66.80/100,000 inhabitants and 12.30/100,000 for the area outside these clusters (rate ratio 5.43). These findings indicate that leprosy is endemic to concentrated in a small portion of the population in Brazil.

**Table 1:** Ten most probable clusters of leprosy defined by using spatial scan statistics, Brazil, 2005–2007*

Cluster order	No. cases		RR	LLR
	Observed	Expected		
1	24,564	6,345.04	4.59	16,545.44
2	9,735	2,224.77	4.67	7,099.49
3	4,136	928.37	4.58	3,014.57
4	6,944	2,912.92	2.47	2,070.23
5	5,778	2,424.91	2.45	1,711.11
6	5,891	2,674.40	2.26	1,479.21
7	2,223	1,039.11	2.16	512.49
8	1,325	476.37	2.80	509.78
9	3,288	1,799.11	1.85	502.97
10	1,473	581.84	2.55	480.32

*RR, relative risk for the cluster compared with the rest of the country; LLR, log likelihood ratio. p<0.001 for all comparisons.

**Figure:**
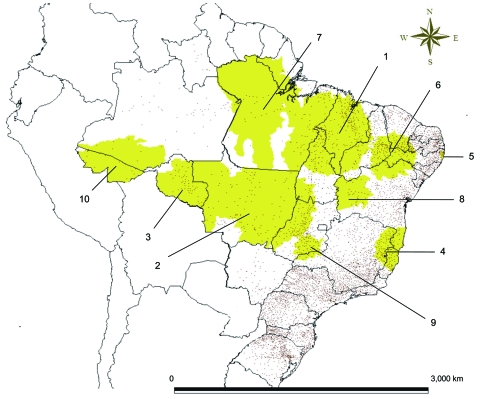
Locations of the 10 most probable leprosy clusters (yellow regions) and municipal councils (dots), Brazil, 2005–2007.

Case-detection rates for each cluster are shown in Table 2. By comparison, the highest case-detection rates reported to the World Health Organization in 2006 were from Micronesia (136.04/100,000, population 602,000) and Papua New Guinea (63.95/100,000, population 111,000) (*8*). Cluster 1, which contained 20.1% of cases detected during 2005–2007, was located in an area that had a population of 9,592,600 in 2007.

**Table 2:** Leprosy case-detection rates and proportion of all cases in the 10 spatial clusters, Brazil, 2005–2007

Cluster order	Case detection rate/ 100,000 inhabitants	% Cases
1	85.36	20.10
2	95.42	7.97
3	97.15	3.39
4	51.99	5.68
5	51.96	4.73
6	48.04	4.82
7	46.65	1.82
8	60.66	1.08
9	39.85	2.69
10	55.21	1.21
Brazil	21.82	100.00

Some municipalities within these clusters had lower case-detection rates than the average case-detection rate in Brazil. A probable explanation is that fewer cases were detected because of failures in the healthcare system, such as low population coverage and the inability of healthcare workers to diagnosis leprosy.

Of the 10 most likely clusters, 5 (1, 2, 3, 7, and 10) were located in the Amazon region of Brazil, and 3 (6, 8, and 9) were contiguous to 1 of these 5 Amazon clusters and located in dry savannah areas. Cluster 5, which was spatially isolated from the others, corresponds to the Recife metropolitan region, the third largest metropolitan area in Brazil. This area is populated by poor immigrants from dry rural areas of northeastern Brazil. Cluster 6, also spatially isolated, was in a region that has a hot, humid climate and contains remnants of the Atlantic rainforest.

## Conclusions

Leprosy is still highly endemic in the Amazon region of Brazil. This fact cannot be of explained by socioeconomic conditions of the population because the populations of northeastern states (semiarid climate) and of large metropolitan areas have a much higher risk for malnutrition than the population in the Amazon region of Brazil.

A hypothesis to explain the clusters in the Amazon region of Brazil is that when leprosy was introduced into this region, it probably caused an epidemic among the indigenous population because of their lack of exposure to infection (*9*). The disease likely spread slowly throughout the area because of the isolated population and large distances. Until 1970, the most heavily populated areas of the Amazon region of Brazil could be described as population islands because the only means of transportation to them were riverboats or small airplanes. For this reason, the epidemiologic pattern of leprosy in this region was similar to that in the Pacific islands, where similar case-detection rates have been noted.

The first highway (length 2,039 km) to cross the Amazon rainforest was completed in 1974. Today, there are 25,900 km of federal highways in the Amazon region of Brazil (*10*). Development projects and small-scale strip mining, mostly for gold, resulted in an increase in the population (*11*). Although agricultural development led to relatively stable settlements in the area, small-scale mining was undertaken by an extremely mobile population. Although the population increase was largely caused by an influx of immigrants from regions of Brazil that have fewer cases of leprosy, this disease remains highly endemic in the Amazon region of Brazil and is now concentrated in the areas of greatest population increase.

Strip miners return seasonally to their homes, and persons involved in unsuccessful land settlement projects often sell their plots and move to new agrarian development projects (*12*). Families who succeeded in land cultivation periodically returned to their city of origin as a sign of success (*13*). This population movement results in leprosy cases in other regions and raises the question whether leprosy can reemerge in other parts of Brazil.

Clusters 6, 8, and 9 are located in dry savannah lands and are adjacent to the Amazon clusters. Since 1980, case-detection rate for leprosy increased in Brazil (*14*), and the northeastern region had the highest increase of any region (*4*). In addition, clusters 6 and 8 must be addressed by the leprosy control program because they overlap current agrarian development initiatives in northeastern Brazil.

## References

[1] Souza Araújo HC. História da lepra no Brasil. Situação da lepra nos estados de 1901 a 1920. Vol. III. Rio de Janeiro (Brazil): Departamento de Imprensa Nacional; 1956.

[2] Cruz OG. 1913 Relatório sobre as condições medico-sanitarias do valle do Amazonas apresentado a Sua Ex o Sr. Dr. Pedro de Toledo–Ministro da Agricultura, Industria e Commercio. In: Oswaldo Gonçalves Cruz: opera omnia. Rio de Janeiro (Brazil): Impr. Brasileira; 1972. p. 663–718.

[3] Agricícola E. Alguns aspectos da epidemiologia e da profilaxia da lepra no Brasil. Revista Brasileira Dermatologia. 1975;50:215–22.

[4] Ministry of Health. Brazil. Health surveillance epidemiological situation of Hansen’s disease in Brazil, 2008. Brasília (Brazil): The Ministry; 2008.

[5] Kulldoff M. A spatial scan statistics. Comm Statist Theory Methods. 1997;26:1481–96. 10.1080/03610929708831995

[6] Dwass M. Modified randomization tests for nonparametric hypotheses. Ann Math Stat. 1957;28:181–7. 10.1214/aoms/1177707045

[7] Kulldorff M and Information Management Services, Inc. Satscan version 7.0. Software for spatial and space-time scan statistics, 2006 [cited 2009 Jan 20]. Available from http://www.satscan.org

[8] World Health Organization. Leprosy global situation [cited 2008 Aug 15]. Available from http://www.who.int/lep/situation/WPROStatsEnd2006.pdf

[9] Tahlhari S, Aguiar AP, Matos TT, Spener S, Borborema CA. Hanseníase no estado do Amazonas—histórico e desativação do leprosário. An Bras Dermatol. 1981;56:179–84.

[10] Sant’Anna JA. Rede básica de transportes da Amazônia. 1998 [cited 2009 Jan 20]. Available from http://www.ipea.gov.br/pub/td/td_562.pdf

[11] Rigotti JI, Vasconcelos IR. Uma análise espacial exploratória dos fluxos populacionais brasileiros nos períodos 1986–1991 e 1995–2000. In: Anais do IV encontro nacional sobre migrações. Rio de Janeiro; Associação Brasiliera de Estudos Populacionais; 2005. p. 1–20.

[12] Becker B. Revisão das políticas de ocupação da Amazônia: é possível identificar modelos para projetar cenários? Parcerias Estratégicas. 2001;12:135–59.

[13] Rocha BN. Em qualquer chão: sempre gaúcho!—A multiterritorialidade do migrante gaúcho no Mato Grosso [master’s dissertation]. Rio de Janeiro (Brazil): Institute of Human and Social Sciences, Federal Rural University of Rio de Janeiro; 2006.

[14] Penna ML, Penna GO. Case detection and leprosy elimination in Brazil. Trop Med Int Health. 2007;12:647–50. 17445132 10.1111/j.1365-3156.2007.01837.x

